# First record of *Orthopodomyia pulcripalpis* (Rondani, 1872) (Diptera: Culicidae) in Austria

**DOI:** 10.1007/s00436-017-5460-8

**Published:** 2017-05-09

**Authors:** Carina Zittra, Adelheid G. Obwaller, Victoria Wimmer, Dominik Berer, Barbara Eigner, Hans-Peter Fuehrer

**Affiliations:** 1 Institute of Parasitology, Department of Pathobiology, University of Veterinary Medicine Vienna, Veterinaerplatz 1, 1210 Vienna, Austria; 2 Federal Ministry of Defence and Sports, Division of Science, Research and Development, Roßauer Laende 1, 1090 Vienna, Austria

**Keywords:** *Orthopodomyia*, Mosquitoes, Species inventory, Austria

## Abstract

During a three-year mosquito monitoring from 2014 to 2016, the strictly ornithophilic, originally Mediterranean species *Orthopodomyia pulcripalpis* (Rondani, 1872) was collected as single specimen for the first time in Austria in the district of Penzing in Vienna. Morphological species determination was confirmed by analysis of the mitochondrial cytochrome c oxidase subunit I gene. We thus not only confirm the existence of another mosquito species in Austria, but also add a new genus to the Austrian Culicidae taxa list.

## Introduction

The genus *Orthopodomyia* comprises 24 dendrolimnocolous species, preferring water-filled bamboo stumps, axils of bromeliads, tree holes or holes in roots of different tree species as breeding sites (Becker et al. 2010). *Orthopodomyia pulcripalpis* is the only species of the genus known from the Palaeartic. The taxon was reported from the Mediterranean region, areas of North-Western Europe, Black Sea cost, Asia Minor, Georgia and Azerbaijan in Transcaucasia (Ramsdale and Snow 2001). Specifically, populations of *Or*. *pulcripalpis* are known from Hungary (Kenyeres and Toth 2012) and doubtfully Switzerland (Fouque et al. 1991; Briegel 1998), bordering Austria. A detailed list of species records in Europe is listed in Ramsdale and Snow (2001), missing only one new record of *Or*. *pulcripalpis* from Belgium (cf. Boukraa et al. 2015). No records of this taxon are so far available from Austria, Germany, Slovenia and the Czech Republic.

*Or*. *pulcripalpis* is a polycyclic species, described to hibernate in the fourth larval instar with the capacity to survive under frozen water surfaces (Becker et al. 2010; Marshall 1938). The species mainly breeds in highly alkaline water bodies which are available in tree holes or holes in roots of different tree species such as *Aesculus hippocastanum*, *Olea europaea*, *Fagus sylvatica*, *Ulmus* spp., *Platanus* spp. and *Quercus* spp. (Munstermann et al. 1985; Cranston et al. 1987; Becker et al. 2010), but of these, permanently water-filled breeding sites are preferred (Becker et al. 2010). Larvae of this species are often found in co-occurrence with larvae of *Anopheles plumbeus*, *Ochlerotatus* *geniculatus*, *Ochlerotatus echinus*, *Ochlerotatus pulcritarsis* and *Ochlerotatus berlandi* (Snow and Medlock 2008; Becker et al. 2010). These species also use water-filled tree holes as larval breeding habitats, but only *Oc*. *geniculatus* and *An*. *plumbeus* are widely distributed in Austria, and mainly breed in tree holes of the common hornbeam (*Carpinus betulus*) (Zittra and Waringer 2015).

Females of *Or*. *pulcripalpis* were described as being ornithophilic, to rarely bite humans, and to be most active during the day in shaded places (Cranston et al. 1987; Ribeiro et al. 1988). Although vector capacity and vector competence of *Orthopodomyia* are particularly poorly known, the genus is assumed to play a role in the distribution of avian arboviruses due to its reported host preference (Zavortink 1968; Becker et al. 2010).

## Material and methods

Mosquito communities were investigated across three provinces of Eastern Austria (Burgenland, Lower Austria and Vienna) within a mosquito surveillance programme at 35 permanent and 23 non-permanent trapping sites. Mosquitoes were monitored on a regular basis every second week for a 24-h time period from April to October 2014–2016 using Biogents Sentinel Traps (Regensburg, Germany) equipped with carbon dioxide as attractant. Non-permanent sampling sites were investigated at least once and up to six times during the summer months.

The collected mosquitoes were stored at −80 °C and morphologically identified using the identification keys of Becker et al. (2010) and Mohrig (1969). Species determination was confirmed by analysis of the mitochondrial cytochrome oxidase subunit I gene (CO1) (KY608735). Genomic DNA was extracted from three legs of the specimen using the DNeasy™ Blood and Tissue Kit (Qiagen, Hilden, Germany) according to the manufacturer’s protocol. Amplification of a ~ 700-bp-long mtCO1 fragment was achieved using primers H15Culi-COIFw and H15CuliCOIRv in standard PCR protocols (Werblow et al. 2015; Zittra et al. 2016). Finally, purified PCR products were sequenced by a commercial company (LGC Genomics GmbH, Germany).

## Results and discussion

Despite extensive adult mosquito monitoring, only a single specimen of *Or*. *pulcripalpis* was collected at the end of August in Penzing, the 14th district of Vienna, at a non-permanent sampling site. While never confirmed previously (Lebl et al. 2015), this species was assumed to exist in Austria (Aspöck, personal communication) due to adequate climatic conditions and the ample availability of suitable breeding sites, e.g. in *C*. *betulus* forests. The sampling site itself and the surrounding area provide abundant breeding habitats for *Or*. *pulcripalpis*, in particular in parks and allotment gardens that comprise stocks of old trees.

Although this species was often described to be rare, it is possible that it was overlooked due to its ornithophilic behaviour and the consequently low attractiveness of commonly used trapping methods with carbon dioxide as main attractant. Indeed, ornithophilic taxa surveillance of adult mosquitoes using only one trapping system can lead to an underestimation of a species abundance as it was reported for, e.g. *Culex torrentium* (Weitzel et al. 2011; Zittra et al. 2016) and *Culiseta longiareolata* (Zittra et al. 2014). Therefore, larval sampling of different aquatic habitat types should be included in standard mosquito monitoring schemes in order to gain more information on the real abundance of strictly ornithophilic species.

The finding of *Or*. *pulcripalpis* in Vienna leads not only to an increase of the Austrian Culicidae species list, but also results in the registration of a new genus in the country. Interestingly, *Oc*. *pulcripalpis* is an expansive Holomediterranean faunal element of the arboreal (Aspöck 2008), which probably survived the latest glacial period (ca. 115,000 to 12,000 BP) in refugial areas in many parts of the Mediterranean region and possibly also in a few scattered extramediterranean refugial areas south of the alps (Aspöck 2008; Aspöck et al. 1991). These refugial areas provided optimal ecological conditions for the development of forests and the basis of survival for this mosquito species (Aspöck 2008; Aspöck 2010). After the glacial period, especially during the Holocene climatic optimum (or Atlanticum), about 6500 BP and about 4500 years BP, this species spreads gradually northwards from different refugial areas, when Northern and Central Europe became forested (Aspöck 2008). However, it likely disappeared again with decreasing temperature in some parts of its distribution area, until postglacial climate allowed propagation (Aspöck 2008). This postglacial spread is probably polycentric, originating from different refugial centres (e.g. the Atlantomediterranean and the Pontomediterranean centres) that were separated one from another since the last interglacial period (De Lattin 1967). Consequently, we assume distinct genetic differences to have evolved between *Or*. *pulcripalpis* populations originating from different refugial centres. As the vector capacity of *Or*. *pulcripalpis* is not well studied, phylogeography, abundance, seasonality, distribution patterns as well as the pathogen load of this rare species need to be investigated in detail.
